# Alteration of circulating unconventional T cells in cerebral ischemia: an observational study

**DOI:** 10.1038/s41598-022-14343-2

**Published:** 2022-06-16

**Authors:** Chao Zhou, Wei Rao, Xinhua Zhou, Dan He, Zhen Li, Nyambayar Dashtsoodol, Yue Ren

**Affiliations:** 1 Department of Neurology, Jiangxi Provincial People’s Hospital, The First Affiliated Hospital of Nanchang Medical College, Nanchang, Jiangxi China; 2 The Neurological Institute of Jiangxi Province, Jiangxi Provincial People’s Hospital, The First Affiliated Hospital of Nanchang Medical College, Nanchang, Jiangxi China; 3 Department of Immunology and Stem Cell Biology, Faculty of Medicine, Institute of Medical, Pharmaceutical and Health Sciences, Kanazawa University, Kanazawa, Ishikawa Japan; 4 Department of Immunology, School of Biomedicine, Mongolian National University of Medical Sciences, Ulaanbaatar, Mongolia; 5 Department of Hematology and Medical Oncology, Klinikum Rechts der Isar and TranslaTUM Cancer Center, Technische Universität München, Munich, Germany

**Keywords:** Immunology, Biomarkers, Neurology

## Abstract

Immune reactions provoked by cerebral ischemia play crucial roles in the pathogenesis of brain damage and contribute to tissue regeneration processes. While functions of many immune cell types in post-ischemic inflammation have been well studied in experimental stroke, the exact roles played by unconventional T cells in pathogenesis of the clinical stroke remain to be precisely determined. In the present study, we investigated the frequencies and absolute cell numbers of peripheral blood T lymphocyte subpopulations including those of invariant natural killer T (iNKT) cells, CD3^+^CD56^+^ NKT-like (NKTL) cells, and γδ T cells from patients with acute cerebral infarction (ACI), chronic cerebrovascular disease (CCD) or chronic cerebral circulation insufficiency (CCI) by flow cytometry, and analyzed their association with the disease severity and the clinical outcome. We observed significantly reduced cell numbers of circulating iNKT cells, NKTL cells and γδ T cells in cerebral ischemia patients as compared with the healthy controls. Of note, we also demonstrated that numbers of peripheral blood iNKT and γδ T cells are significantly reduced in patients with ACI when compared among different cerebral ischemia patient groups. Moreover, the reduced number of iNKT cells is significantly associated with the disease severity and recovery in cerebral ischemia patients. Our results demonstrate for the first time the reduction of peripheral blood NKTL, iNKT and γδ T cells in patients with the cerebral ischemia, and particularly reduced iNKT and γδ T cells in the acute phase. The reduction of iNKT cells seems to be significantly associated with the disease severity and recovery. We hope that our findings might lead to the identification of predictive and prognostic values of human peripheral unconventional T cell subsets in the cerebral ischemia.

## Introduction

Ischemic stroke (IS) is one of the leading causes of death and disability worldwide, with a high incidence and recurrence rate in aged population^1^. Although tremendous efforts have been elaborated in attempt to understand the pathologic processes underlying the IS, it remains to be thoroughly investigated regarding the prevention, early diagnosis, and treatment of IS.

Immune reactions followed by cerebral ischemia play a critical role in the pathogenesis of tissue damage caused by IS. To date number of studies on murine models of the experimental cerebral ischemia have described different lymphocyte subpopulations that play diverse roles during the pathogenesis of IS^2,3^. It is reported that changes in composition and numbers of immune cells are not only present in brain, but also observed in peripheral sites during IS^4^. Activation and re-distribution of immune cells occurs shortly after the cerebral ischemia. Microglia in the central nervous system (CNS) and peripheral immune cells, including monocytes/macrophages, neutrophils and lymphocytes, are recruited into the ischemic cerebral hemisphere that are thought to induce local inflammatory responses^5,6^. It is now well accepted that the peripheral immune cells infiltrating the post-ischemic brain play an essential role in pathophysiology of stroke and contribute to secondary neuro-degeneration^7,8^. The long-lasting immunological communication between brain and periphery seems to result in extremely complex effects on the progression of ischemic brain injury and the following tissue regeneration processes^9,10^. It is reported that among various T cell populations recruited to the stroke brain in addition to the conventional CD4 T cells^11^ and CD8 T cells^12^, the unconventional T lymphocytes such as γδ T cells^13^, regulatory T cells (Treg)^14^, as well as natural killer T (NKT) cells^15^ play important roles in IS pathology. While functions of peripheral immune cells in post-ischemic inflammation were well studied using animal models of the experimental stroke, their roles in the pathology of the clinical stroke remain to be investigated.

Alterations in the composition of peripheral immune cell subsets have been reported in several central nervous system (CNS) disorders, such as multiple sclerosis^16^, Parkinson’s Disease^17^, as well as spinal cord injury^18^. These changes usually correlate with disease severity and are considered to be predictive for above-mentioned pathologies. Acute ischemic stroke (AIS) was reported to correlate with the peripheral immune depression^19,20^, and the stroke prognosis and outcomes were also linked to changes of circulating peripheral lymphocyte cell numbers^21,22^. However, it should be noted that the limitation of most of studies was a small sample size and mainly focused investigation on the AIS. In addition, although changes of conventional T cell subsets during the clinical stroke were well-studied, it still needs to be investigated regarding alterations of unconventional T cell subpopulations in peripheral blood of stroke patients and their association with disease outcomes.

In the present study, we assessed the frequencies and absolute cell numbers of unconventional T cell subpopulations, including invariant NKT (iNKT) cells, CD3^+^CD56^+^ NKT-like (NKTL) cells, and γδ T cells in the peripheral blood of patients with acute cerebral infarction (ACI), chronic cerebrovascular disease (CCD), and chronic cerebral circulation insufficiency (CCI), and analyzed their association with the disease severity and outcomes. Our findings will hopefully provide evidence that could be useful in identifying predictive and prognostic values of peripheral immune cells in the clinical cerebral ischemia.

## Methods

### Study subjects

Study participants were consecutively recruited from August 2017 to May 2019 from inpatients in the Department of Neurology who were diagnosed with acute cerebral infarction (ACI, patients present with acute ischemic lesion), chronic cerebrovascular disease (CCD, patients present with large cerebral arteries stenosis with history of ACI, course of disease ≥ 3 months), or chronic cerebral circulation insufficiency (CCI, patients present with large cerebral arteries stenosis without history of ACI, course of disease ≥ 3 months), and the healthy controls (HC) who underwent a routine health checkup procedure at the Jiangxi Provincial People’s Hospital Affiliated to Nanchang University, China. Cerebral ischemia was diagnosed according to World Health Organization stroke criteria. National Institutes of Health Stroke Scale (NIHSS)^23^ and Barthel Index scores^24^ were recorded on patient admission and discharge. The exclusion criteria were: (1) age ≥ 80; (2) severe heart and lung dysfunction; (3) allergies; (4) known inflammatory disease or systemic infections; (5) cancer; (6) hematology diseases; (7) immune compromised diseases; (8) diseases of the connective tissue; (9) undergoing steroid or immunosuppressive therapies. The study protocol was approved by the ethics committee of the Jiangxi Provincial People’s Hospital Affiliated to Nanchang University, China. All participants gave fully informed consent directly or through a surrogate when appropriate.

### Flow cytometry

The peripheral blood samples were collected in EDTA from patients between 6:00 AM and 7:00 AM on day 1 after admission. After collection, blood samples were incubated with fluorescence labeled monoclonal antibodies to define immune cell subpopulations. Antibodies (BD Biosciences or BioLegend) used for flow cytometry detection were: PE anti-CD3, APC anti-TCR Vα24-Jα18, APC anti-CD56, FITC anti-TCR γδ, Percp anti-CD19. Samples were analyzed with Mindray BriCyte E6 (Mindray). Immune cell subpopulations analyzed in this study were defined as follows: T cells (CD3^+^, CD19^−^), iNKT cells (CD3^+^, TCR Vα24-Jα18^+^), NKTL cells (CD3^+^, CD56^+^), γδ T cells (CD3^+^, TCR γ/δ^+^) (Supplementary Fig. 1).

### Statistical analyses

Data are presented as mean ± SD, or median [range]. Non-parametric and parametric tests, as appropriate, were used to compare demographic and laboratory values between the patients and HC subjects or among patient groups (Wilcoxon rank sum, Student’s t tests, or ANOVA). The ordered logistic regression adjusting for subjects’ age and sex was performed to test the association of cell subsets with disease severity measured by NIHSS and Barthel Index scores. As the cell count and percentage were highly correlated, we have performed the regression separately for the cell count and percentage. *P* < 0.05 is considered statistically significant. Data were cleaned using R version 4.1.0. Analyses were performed using R version 4.1.0.

### Ethics approval and consent to participate

All the subjects provided the written consent, and the study protocol was in accordance to the Declaration of Helsinki and was approved by the institution review board (IRB) of Jiangxi Provincial People’s Hospital Affiliated to Nanchang University, Nanchang, China.

## Results

### Characteristics of study participants

A total of 254 cerebral ischemia patients, including 114 with acute cerebral infarction (ACI), 106 with chronic cerebrovascular disease (CCD), and 34 with chronic cerebral circulation insufficiency (CCI), as well as 62 healthy controls (HC) were recruited in the present study. The demographic and clinical characteristics of all study participants are summarized in Table 1. The mean age of patient and HC group were 61.9 ± 11.2 and 59.4 ± 5.6 years old respectively. 42.1% of patients were female, while the percentage of female participants in HC group was 51.6%. There was no significant difference in age and gender between patient and HC groups (*P* > 0.05). Among the patients, the mean age of ACI, CCD, and CCI groups were 62.0 ± 11.3, 64.4 ± 9.9 and 53.4 ± 10.7 years old, respectively, where significant differences among the three stroke groups were observed (*P* < 0.05). Of note, the CCI group had the lowest mean age and significantly lower than ACI and CCD groups (both *P* < 0.05), however there is no significant difference between ACI and CCD group in age (*P* > 0.05). Significant differences in gender among the three stroke groups were also observed (*P* < 0.05), of which only significance between ACI and CCD group was detected. There was no significant difference in the prevalence of comorbidities between patient and HC groups (*P* > 0.05). Among the three patient groups, significant differences in smoking, hypertension, and diabetes were detected (*P* < 0.05) (Table 1).

**Table 1:** Characteristics of the study cohort.

	Patients				Total patients (n = 254)	HC (n = 62)	*P*^*b*^
	ACI (n = 114)	CCD (n = 106)	CCI (n = 34)	*P*^*a*^			
**Demographics**							
Age	62.0, 11.3	64.4, 9.9	53.4, 10.7	** < 0.001**	61.9, 11.2	59.4, 5.6	0.096
Sex (Female %)	38 (33.3%)	54 (50.9%)	15 (44.1%)	**0.029**	107 (42.1%)	32 (51.6%)	0.200
**Stroke scores at admission**							
NIHSS	5 [0–35]	0 [0–12]	0 [0–8]	** < 0.001**	2 [0–35]	/	/
Barthel	80 [0–100]	100 [40–100]	100 [50–100]	** < 0.001**	100 [10–100]	/	/
**Stroke scores at discharge**							
NIHSS	2 [0–35]	0 [0–7]	0 [0–3]	** < 0.001**	0 [0–35]	/	/
Barthel	90 [0–100]	100 [50–100]	100 [95–100]	** < 0.001**	100 [10–100]	/	/
**Comorbidities (N, %)**							
Smoking	47 (41.2%)	27 (25.5%)	11 (32.4%)	**0.046**	83 (32.7%)	19 (31%)	0.443
Hypertension	79 (69.3%)	64 (60.4%)	12 (35.3%)	**0.002**	155 (61.0%)	32 (52%)	0.114
Hyperlipidemia	60 (52.6%)	48 (45.3%)	10 (29.4%)	0.056	118 (46.5%)	27 (44%)	0.395
Diabetes	39 (34.2%)	14 (13.2%)	3 (8.8%)	** < 0.001**	56 (22.0%)	8 (13%)	0.072
Coronary artery disease	6 (5.3%)	11 (10.4%)	1 (2.9%)	0.201	18 (7.1%)	2 (3%)	0.209
Hyperhomocysteinemia	12 (10.5%)	6 (5.7%)	1 (2.9%)	0.218	19 (7.5%)	4 (6%)	0.517
Atrial fibrillation	7 (6.1%)	5 (4.7%)	1 (2.9%)	0.736	13 (5.1%)	0 (0%)	0.055

Significant values are in bold.

*P*^*a*^: *P* values from comparisons among 3 groups of ischemic patients. *P*^*b*^: *P* values from comparisons of all patients and the healthy control group. Data were presented as mean, SD, or median [range]. *ACI* acute cerebral infarction, *CCD* chronic cerebrovascular disease, *CCI* chronic cerebral circulation insufficiency, *HC* healthy control, *NIHSS* National Institutes of Health Stroke Scale.

Clinical severity of stroke was measured using the National Institutes of Health Stroke Scale (NIHSS) and Barthel Index. The NIHSS scores of ACI group at admission and discharge were 5(0–35) and 2(0–35), respectively, which were significantly higher than those of CCD and CCI groups (*P* < 0.05). Lower Barthel index was also recorded in ACI group, both at the admission and discharge (80(0–100) and 90(0–100), respectively) (*P* < 0.05) (Table 1).

### Profiling of peripheral T lymphocyte subsets in patients with cerebral ischemia

Frequencies and absolute numbers of peripheral blood immune cell subsets from patients and HC group were analyzed by flow cytometry. We compared the cell counts and proportions of peripheral T cell subset between patients and control individuals. As shown in Table 2, cerebral ischemia patients showed significantly reduced cell numbers and percentages of γδ T cells, NKTL cells and iNKT cells (*P* < 0.046), while there was no significant difference in the cell number and proportion of total T lymphocytes between patients and HC groups (*P* > 0.05) (Table 2).

**Table 2:** Profiles of peripheral immune cell subsets in patients and healthy controls.

	Patients				Total patients (n = 254)	HC (n = 62)	*P*^*b*^
	ACI (n = 114)	CCD (n = 106)	CCI (n = 34)	*P1/P2/P3*^*a*^			
**Cell counts (count/μL)**							
Lymphocyte	1359, 522	1463, 461	1343, 507	0.225/0.879/0.197	1400, 496	1522, 538	0.092
T	856, 349	933, 337	876, 377	0.253/0.772/0.407	891, 348	957, 363	0.194
γδT	39, 28	61, 70	44, 36	**0.004**/0.368/0.165	49, 52	80, 94	**0.016**
iNKT (/mL)	722, 763	1039, 1373	1600, 2916	**0.009**/**0.004**/0.129	972, 1495	1690, 2526	**0.034**
NKT-like	50, 44	64, 66	51, 46	0.132/0.918/0.274	56, 55	90, 82	**0.002**
**Cell proportion**							
T (%lym)	67.5, 10.6	68.7, 8.8	69.2, 9.4	0.558/0.409/0.775	68.2, 9.7	66.8, 8.7	0.265
γδT (%T)	4.3, 2.6	6.0, 6.0	4.9, 4.4	**0.02**/0.345/0.306	5.1, 4.6	7.9, 8.1	**0.011**
iNKT (%T)	0.09, 0.10	0.11, 0.17	0.17, 0.23	0.051/**0.009**/0.152	0.11, 0.15	0.18, 0.27	**0.046**
NKT-like (%T)	5.4, 4.0	6.3, 5.9	5.7, 5.1	0.434/0.663/0.651	5.8, 5.0	9.1, 6.2	** < 0.001**

Significant values are in bold.

^a^*P* values from comparisons within ischemic groups. *P1*, ACI vs CCD; *P2*, ACI vs CCI; *P3*, CCD vs CCI. b*P* values from comparisons of all patients and the healthy control group. Data were presented as Mean, SD. *ACI* acute cerebral infarction, *CCD* chronic cerebrovascular disease, *CCI* chronic cerebral circulation insufficiency, *HC* healthy control, *lym* lymphocyte.

Interestingly, when peripheral T cell subpopulations were compared among the three patient groups with different phases of the cerebral ischemia, we observed significantly decreased iNKT cell counts (*P* = 0.009 vs CCD, and *P* = 0.004 vs CCI) (Table 2, Supplementary Fig. 2a) and percentages (*P* = 0.051 vs CCD, and *P* = 0.009 vs CCI) (Table 2, Supplementary Fig. 2b) in the ACI group compared to both CCD and CCI groups. Furthermore, the ACI patient group had significantly reduced numbers (*P* = 0.004) and frequencies (*P* = 0.02) of γδ T cells compared to the CCD group (Table 2). Besides the above-mentioned differences, the cell counts and frequencies of total T lymphocytes and NKTL cells were comparable among the three patient groups.

### Correlation of peripheral iNKT and γδ T cells with ACI and the clinical severity of cerebral ischemia

Since we observed significantly decreased iNKT and γδ T cells in patients with ACI when compared among patient groups, however, significant differences in age, gender, as well as comorbidities including smoking, hypertension and diabetes were also observed among patient groups. In order to clarify whether iNKT and γδ T cells are associated with the acute phase of cerebral ischemia, we firstly performed linear regression analysis to test the association of age, gender, as well as comorbidities (smoking, hypertension and diabetes) with iNKT and γδ T cells in all of patients. We found no correlation of iNKT and γδ T cells with smoking, hypertension and diabetes (*P* > 0.103), negative correlation of iNKT cells with age (*P* < 0.01), and positive correlation of γδ T cells with gender (female) (*P* < 0.05). Therefore, we next tested the association of iNKT and γδ T cells with the acute phase of cerebral ischemia using an ordered logistic regression model with the age and sex adjustments to exclude the influence of these factors. As shown in Table 3, the cell counts of iNKT and γδ T cells as well as γδ T cell frequencies were found to be negatively correlated with ACI in all patients with cerebral ischemia.

**Table 3:** Correlation of peripheral blood iNKT and γδT cells with acute cerebral infarction in patients.

	Coefficient	SE	*P* value
iNKT#	− 4.85E-05	2.15E−05	0.025
iNKT%	− 0.3003	0.2093	0.153
γδT#	− 0.0015	0.0006	0.014
γδT%	− 0.0140	0.0068	0.039

*%* percentage, *#* cell count, *SE* standard error.

Furthermore, we examined whether peripheral iNKT, γδ T, and NKTL cells are associated with the disease severity of cerebral ischemia using an ordered logistic regression model with the adjustments of age and sex. We found the cell counts of peripheral iNKT cells to be negatively associated with NIHSS scores at admission (Coefficient = − 0.0001, SE = 5.39E−05, *P* = 0.036) and discharge (Coefficient = − 0.0002, SE = 6.01E−05, *P* = 0.000) in all patients with cerebral ischemia. On the contrary, no significant association with NIHSS scores was observed for the rest of peripheral cell subpopulations. In addition, there was also no significant correlation for all of the three T cell subsets with the clinical severity of cerebral ischemia estimated by the Barthel Index.

### Association of iNKT cells with the clinical outcomes of cerebral ischemia

**Figure 1:**
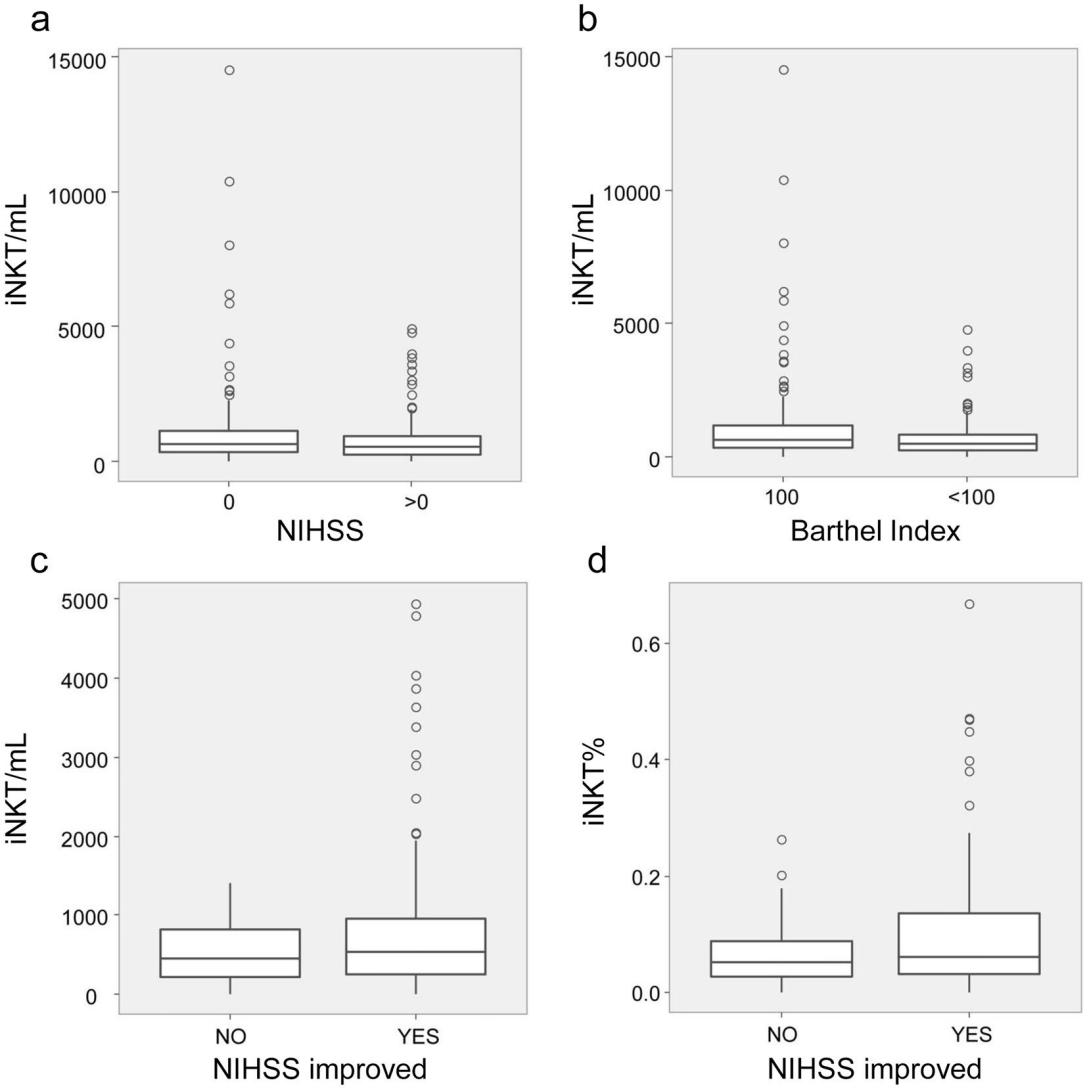
Peripheral blood iNKT cells are decreased in patients with impaired neurological function, activity of daily living and unimproved NIHSS scores. The boxplots of the peripheral blood iNKT cells counts in patients divided by NIHSS score (**a**) or Barthel index (**b**). The boxplots of the peripheral blood iNKT cell counts (**c**) and cell percentages (**d**) in patients grouped according to NIHSS scores at discharge focusing whether their scores were improved or not. NIHSS, National Institutes of Health Stroke Scale.

To further explore the association of iNKT cells with the clinical severity of cerebral ischemia, we compared the numbers and percentages of iNKT cells in patients that were grouped according to presence (NIHSS score > 0) or absence (NIHSS score = 0) of neurological impairment at admission. Results showed significantly decreased iNKT cell counts in patients with impaired neurological functions (NIHSS score > 0) (*P* = 0.028) (Fig. 1a). In addition, when iNKT cell numbers were evaluated in patients grouped by daily living activity measurement at admission (Bathel Index < 100 or = 100), it was found that patients with Bathel Index < 100 had significantly reduced numbers of iNKT cells compared to Bathel Index = 100 patients (*P* = 0.014) (Fig. 1b).

Next, we grouped patients with the NIHSS score > 0 at admission into two groups according to NIHSS scores at discharge focusing whether their scores were improved or not, and then assessed the cell counts and frequencies of iNKT cells between the two patient groups. As shown in Fig. 1c,d, both percentages and absolute numbers of iNKT cells were significantly higher in patients with improved NIHSS scores than those with unimproved scores (*P* = 0.015 and 0.004 for cell frequency and cell count, respectively). These results suggest that the reduced numbers of peripheral iNKT cells in cerebral ischemia patients might be associated with the disease severity and recovery.

## Discussion

Post-ischemic immune reactions play essential roles in the progression of ischemic brain injury and tissue regeneration processes^5,9^. It is now generally accepted that T cells contribute to the pathogenesis of many neurological diseases by inducing innate or adaptive immune responses. In particular, unconventional T lymphocyte subsets such as iNKT and γδT cells were shown to play important roles in the immune regulation that act mainly by bridging the innate and acquired immune responses^25,26^. Emerging studies using animal models of the experimental stroke suggest presence of a link between unconventional T lymphocyte subsets and the IS pathology. In this regard, it was reported that γδ T cells contribute to stroke damage by their production of IL-17 in an IL-23-dependent manner in tMCAO^13^, and NKT cells, including iNKT and NKTL cells were found to infiltrate into the ischemic brain of tMCAO and pMCAO mouse models^15,27^. However, it should be noted that precise roles of unconventional T cell subtypes in human cerebral ischemia remain unclear. In the present study we demonstrate that cerebral ischemia patients possess significantly reduced numbers of circulating iNKT cells, NKTL cells and γδ T cells when compared with the healthy controls. Of note, we also demonstrated that peripheral blood iNKT and γδ T cell numbers are significantly reduced in patients with ACI when compared among cerebral ischemia patient groups. Furthermore, peripheral blood iNKT and γδ T cell counts appears to be negatively correlated with ACI, which might imply to the possibility of accumulating of these cells to the damaged areas in the CNS of cerebral ischemia patients. Thus, our results strongly suggest the possible involvement of NKT and γδ T cells in the cerebral ischemia pathogenesis, particularly in the AIS.

Since we observed significant differences in iNKT and γδ T cells among different phases of the cerebral ischemia and since altered numbers and functions of circulating lymphocytes are known to be associated with the stroke outcome^21,22,28^, we have investigated the correlation of peripheral T cell subsets with the clinical severity of the cerebral ischemia. Our results demonstrate that the cell counts of peripheral iNKT cells are negatively associated with the clinical severity of the cerebral ischemia estimated by NIHSS scores at admission and discharge. Moreover, our data indicate that patients with impaired neurological functions and activity of daily living show significantly decreased numbers of iNKT cells (NIHSS score > 0 vs = 0, Bathel Index < 100 vs = 100). We also observed that the patients with unimproved NIHSS scores at discharge have significantly lower numbers and frequencies of peripheral blood iNKT cells than those patients with improved scores at discharge, which suggests that the reduced numbers of peripheral iNKT cells in cerebral ischemia patients might be associated with the disease severity and recovery. Our present results might imply for the possibility of iNKT cells to be used as a monitoring or prediction index for the treatment of the cerebral ischemia patients.

As iNKT cells are known to directly regulate immune responses through their rapid and massive production of a wide range of cytokines, or indirectly through their regulation of other immune cell types, and as iNKT cells can be further divided into functional subgroups based on their cytokine producing characteristics^25^, the precise role of iNKT cells in cerebral ischemia could be immensely complex and diverse. Some conflicting results derived from animal experimental models were reported regarding the role of iNKT cells on severity of the cerebral stroke. Using pMCAO mouse model, Wang et al. reported that α-galactosylceramide (α-GalCer) activated iNKT cells contribute to brain infarction through their increased pro-inflammatory cytokine production^15^. In contrast, a minor role for iNKT cells in the brain infarction after tMCAO was reported using CD1d-deficient mouse that lacks both CD1d-dependent Vα14 invariant and variant NKT cells. In addition, it was also reported that the selective production of Th2-type cytokines by NKT cells prevents mice from post-stroke infections^29,30^. To date, although there is scarce information regarding human iNKT cell involvement in the cerebral stroke, it was proposed that activated iNKT cells induced by the stroke might relinquish an immunosuppressive response by releasing the immunosuppressive cytokine IL-10^31^. However, it should be pointed that correlation of iNKT cells with the disease severity was not addressed in the above-mentioned study, and their data on unchanged numbers of circulating iNKT cells seems to be inconsistent with our data demonstrated in the present study. The possible explanation for these seemingly contrary conclusions might be related to the sample size differences, where smaller sample size was applied in the report by Wong et al.^31^, and to the difference in patient selection criteria, where, in contrast to patients selected by Wong et al.^31^, patients with manifestations of infection were excluded from our present study.

As an observational study, our work does not provide mechanistic insights into the roles of unconventional T lymphocyte subpopulations such as iNKT and γδ T cells in the development of cerebral ischemia. Nonetheless, we believe our results warrant the necessity for further investigations aimed to study the precise roles of different functional subsets of iNKT cells or γδ T cells both in human cerebral ischemia and in animal models. We also believe our findings will hopefully stimulate the research area bringing into attention the importance of studying unconventional T-lymphocytes in the pathogenesis of central nervous system disorders.

## Conclusions

In summary, this is the first study to characterize the profiles of circulating unconventional T lymphocyte subpopulations in patients with different phases of cerebral ischemia. Our results demonstrate for the first time the reduction of peripheral blood NKTL, iNKT and γδ T cells in patients with the cerebral ischemia, and particularly reduced iNKT and γδ T cells in the acute phase. The reduction of iNKT cells seems to be significantly associated with the disease severity and recovery in cerebral ischemia patients. In summary, our findings derived from detailed flow cytometric analyses of peripheral blood immune cell subpopulations might provide important insights which could be useful in identifying predictive and prognostic values of the cerebral ischemia.

## Supplementary Information

Supplementary Information.

## Data Availability

All data generated or analysed during this study are included in this published article [and its supplementary information files].

## Abbreviations

iNKT: Invariant natural killer T
NKTL: NKT-like
ACI: Acute cerebral infarction
CCD: Chronic cerebrovascular disease
CCI: Chronic cerebral circulation insufficiency
IS: Ischemic stroke
HC: Healthy control
NIHSS: National Institutes of Health Stroke Scale
CNS: Central nervous system
α-GalCer: α-Galactosylceramide
tMCAO: Transient middle cerebral artery occlusion
pMCAO: Permanent middle cerebral artery occlusion
