# Supplementary material for: Alteration of circulating unconventional T cells in cerebral ischemia: an observational study
Source: Sci Rep. 2022 Jun 16;12:10078. doi: 10.1038/s41598-022-14343-2 (PMC9203798; doi:10.1038/s41598-022-14343-2)
Supplement: Supplementary file 1 — Supplementary Information. [file 41598_2022_14343_MOESM1_ESM.docx]

# Alteration of Circulating Unconventional T Cells in Cerebral Ischemia: An Observational Study

**Chao Zhou ^1^, Wei Rao ^1^, Xinhua Zhou ^1^, Dan He ^2^, Zhen Li ^2^, Nyambayar Dashtsoodol ^3, 4, 5^, and Yue Ren ^2^***

^1^ Department of Neurology, Jiangxi Provincial People’s Hospital, The First Affiliated Hospital of Nanchang Medical College, Nanchang, Jiangxi, China.

^2^ The Neurological Institute of Jiangxi Province, Jiangxi Provincial People’s Hospital, The First Affiliated Hospital of Nanchang Medical College, Nanchang, Jiangxi, China.

^3^ Department of Immunology and Stem Cell Biology, Faculty of Medicine, Institute of Medical, Pharmaceutical and Health Sciences, Kanazawa University, Kanazawa, Ishikawa, Japan.

^4^ Department of Immunology, School of Biomedicine, Mongolian National University of Medical Sciences, Ulaanbaatar, Mongolia.

^5^ Department of Hematology and Medical Oncology, Klinikum rechts der Isar and TranslaTUM Cancer Center, Technische Universität München, München, Germany.

* **Correspondence:**

Yue Ren, Email: [reny427@hotmail.com](mailto:reny427@hotmail.com);

Supplementary Material


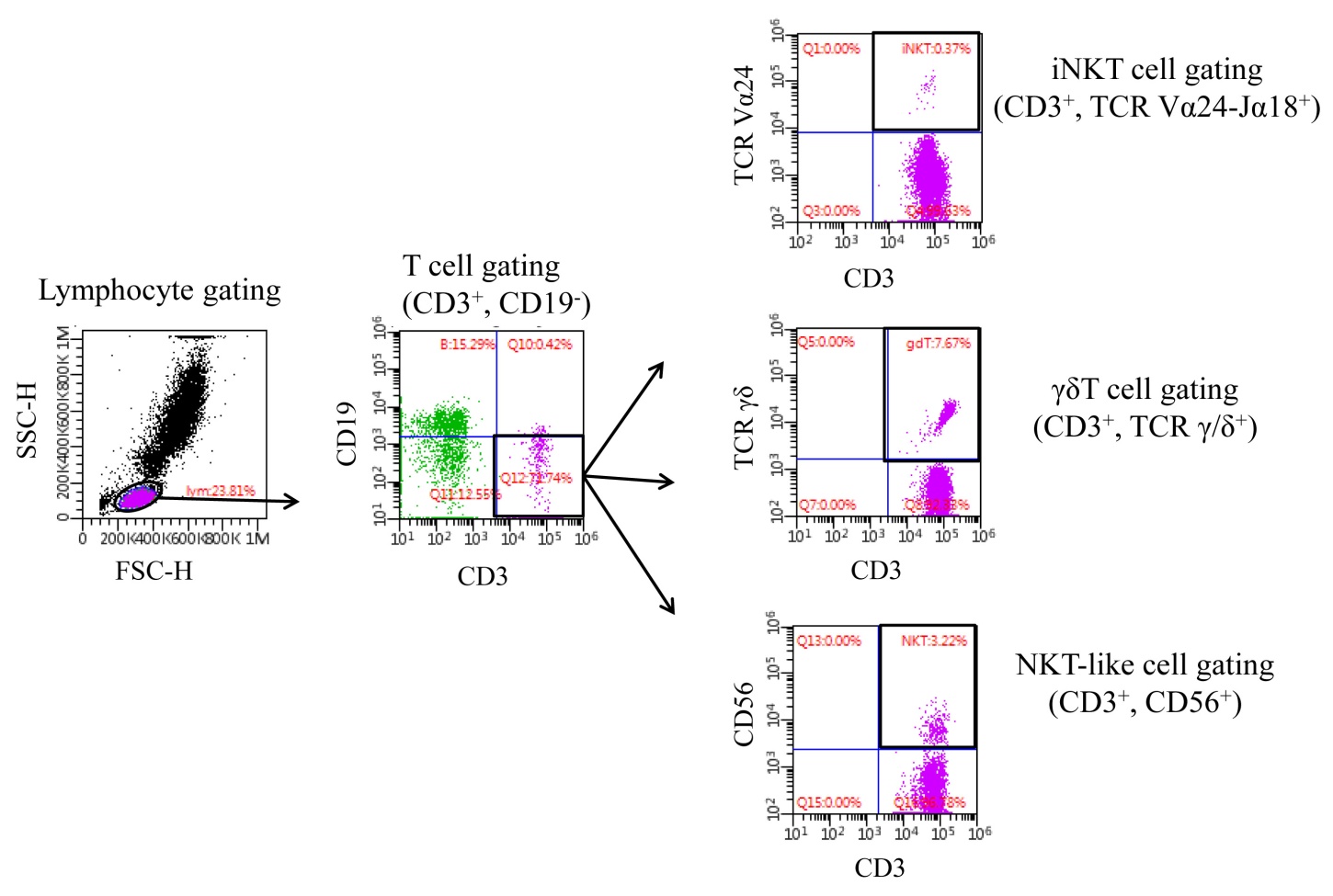


**Supplementary Figure 1.** **Gating strategy to identify T cell subsets.** Lymphocytes were gated based on their forward and side scatter characteristics. T cells were identified by their CD3 and CD19 expression pattern within the gated lymphocyte population. Within T cell population, iNKT, γδ T, and NKT-like cells were further identified based on the differential expression of TCR Vα24-Jα18, TCR γ/δ, and CD56, respectively.


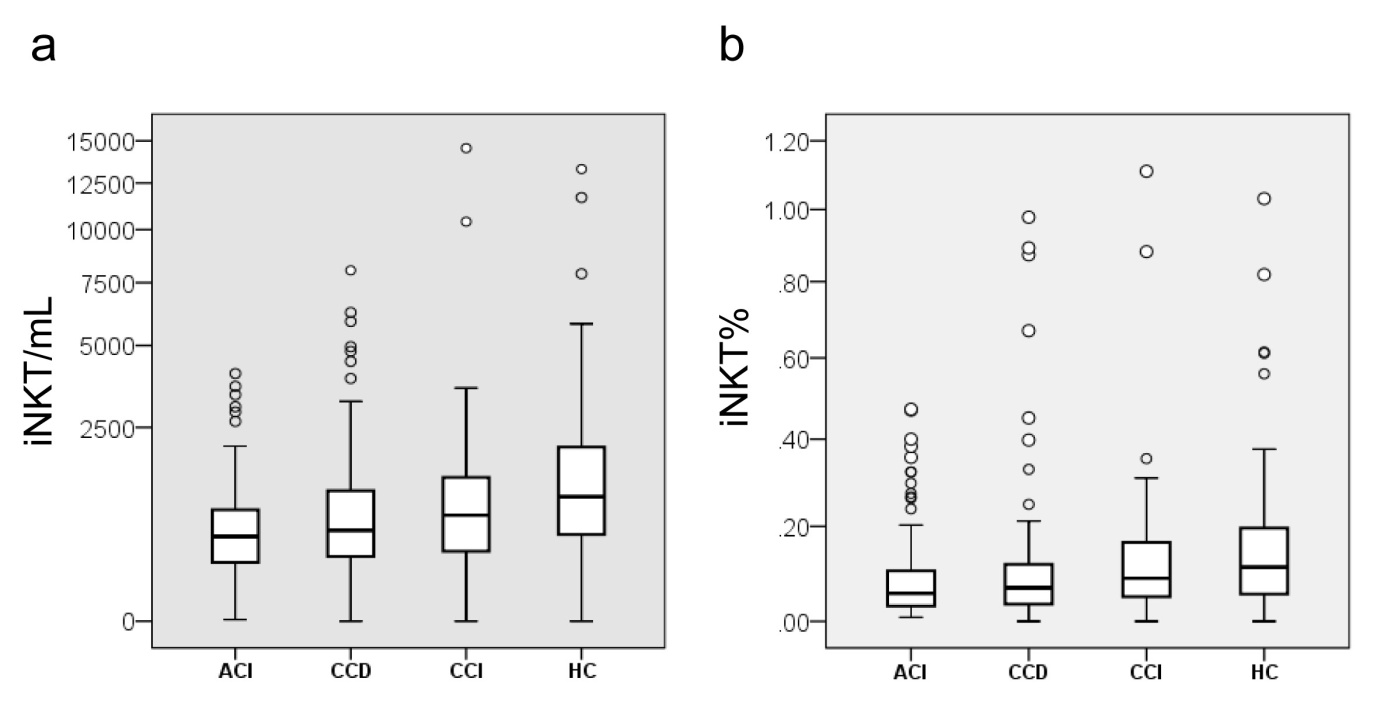


**Supplementary Figure 2.** **Peripheral blood iNKT cells in patients and healthy controls.** The boxplots of the peripheral blood iNKT cell counts (a) and cell percentages (b). ACI, acute cerebral infarction; CCD, chronic cerebrovascular disease; CCI, chronic cerebral circulation insufficiency; HC, healthy control.
